# Supplementary material for: Segment-Specific Functional Responses of Swine Intestine to Time-Restricted Feeding Regime
Source: Animals (Basel). 2025 Dec 24;16(1):52. doi: 10.3390/ani16010052 (PMC12784904; doi:10.3390/ani16010052)
Supplement: Supplementary file 1 [file animals-16-00052-s001.zip › animals-4009209-supplementary.pdf]

Table S1 Ingredients composition and calculated nutritional level of the experiment diet (as-fed basis).

Ingredient	Percentage (%)	Calculated nutritional compositions (%)	
Corn	70.0	Digestive energy（MJ/kg）	14.60
Soybean meal	18.0	Crude protein	16.00
Wheat bran	6.50	Lysine	1.23
Soybean oil	1.90	Methionine+Cystine	0.70
Lysine	0.69	Threonine	0.79
Methionine	0.24	Tryptophan	0.22
Threonine	0.30		
Tryptophan	0.07		
Calcium hydrogen phosphate	0.45		
Stone powder	0.50		
Salt	0.30		
Multivitamins[1]	0.03		
Minerals[2]	0.20		
Choline chloride (50%)	0.12		
Zeolite powder	0.60		
Antioxidant	0.1		
Total	100.0		

[1] The minerals supply per kg diet as follows: Fe 165 mg, Zn 165 mg, Cu 16.5 mg, Mn 30 mg, Co 0.15 mg, I 0.25 mg, Se 0.25 mg.

[2] The multivitamins supply per kg diet as follows: VA 11 000 IU, VD3 1 000 IU, VE 16 IU, VK1 1mg, VB1 0.6 mg, VB2 0.6 mg, d-pantothenic acid 6 mg, nicotinic acid 10 mg, VB12 0.03 mg, folic acid 0.8 mg, VB6 1.5 mg.

Table S2 Summary of RNA-sequencing data

Treatment	Jejunal mucosa			Colonic mucosa		
	Total Reads	Mapped Reads	Alignment rate (%)	Total Reads	Mapped Reads	Alignment rate (%)
FA1	47299552	44680416	94.46	46767998	44256454	94.63
FA2	43706824	41290850	94.47	54093458	51088216	94.44
FA3	44138988	41550034	94.13	59649512	56329288	94.43
FA4	45028740	42247449	93.82	45454880	42925204	94.43
TRF1	50795826	47925373	94.35	45916750	43479377	94.69
TRF2	43878954	41355099	94.25	46984826	43963554	93.57
TRF3	51195538	48391425	94.52	45721202	42889635	93.81
TRF4	47677460	44745625	93.85	50712452	47474608	93.62

FA = free access; TRF = time-restricted feeding
