# Supplementary figures and images for: Segment-Specific Functional Responses of Swine Intestine to Time-Restricted Feeding Regime
Source: Animals (Basel). 2025 Dec 24;16(1):52. doi: 10.3390/ani16010052 (PMC12784904; doi:10.3390/ani16010052)

## Duodenum

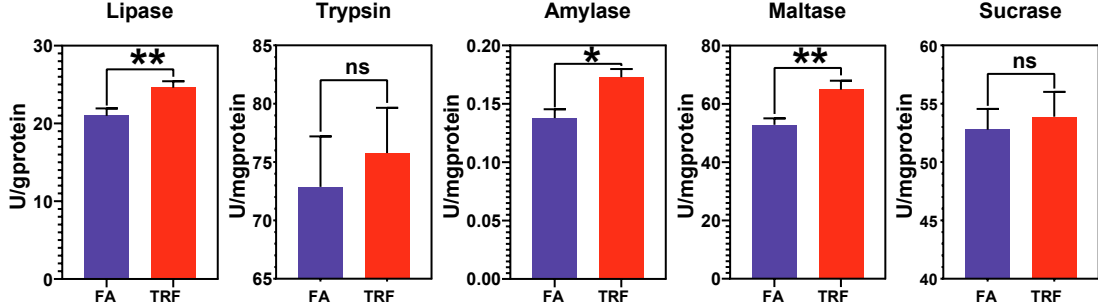

## Pancreas

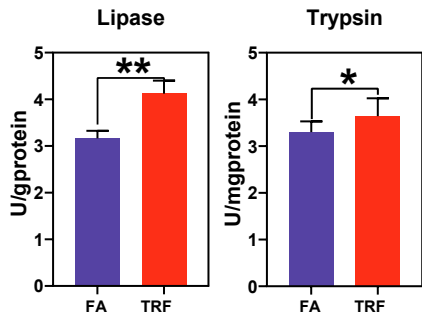

Supplement: Supplementary file 1 [file animals-16-00052-s001.zip › Figure S1.pdf]
